# Supplementary material for: Counting with DNA in metabarcoding studies: How should we convert sequence reads to dietary data?
Source: Mol Ecol. 2018 Jun 17;28(2):391–406. doi: 10.1111/mec.14734 (PMC6905394; doi:10.1111/mec.14734)
Supplement: Supplementary file 1 [file MEC-28-391-s001.docx]

**Supplemental Information for:**

**Counting with DNA in metabarcoding studies: how should we convert sequence reads to dietary data?**

Bruce E. Deagle (corresponding author: Bruce.Deagle@aad.gov.au)

Austen C. Thomas

Julie C. McInnes

Laurence J. Clarke

Eero J. Vesterinen

Elizabeth L. Clare

Tyler R. Kartzinel

J. Paige Eveson

**Table of Contents:**

| **Additional simulation results** | Page 2 |
| --- | --- |
| **Simulation methods** | Page 3 |
| **Table S1. Use of relative read abundance (RRA)**  **and frequency of occurrence (FOO) in studies of bacterial/archaeal communities** | Page 5 |
| **Table S2. Use of relative read abundance (RRA)**  **and frequency of occurrence (FOO) in metabarcoding**  **studies of eukaryote communities** | Page 7 |

## Additional simulation results

(a) Mean of 3 taxa per sample

##
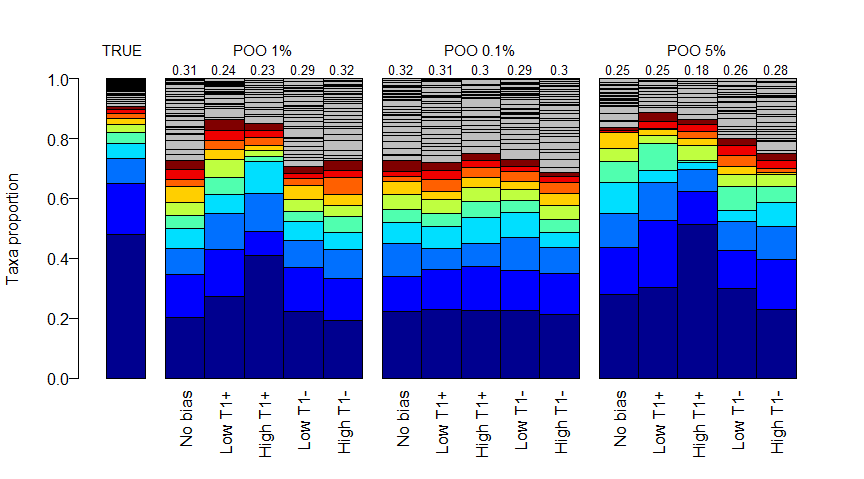


(b) Mean of 20 taxa per sample


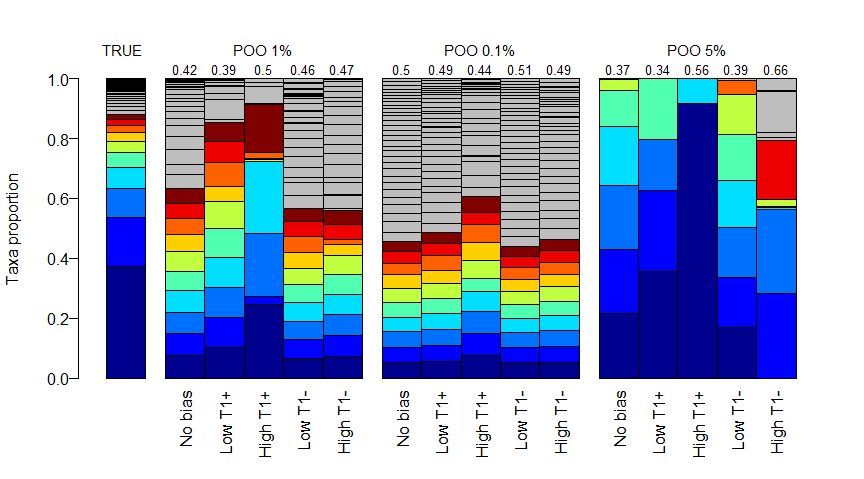


**Figure S1.** Additional simulation results using the occurrence summary (POO), but showing the effect of using a 0.1 or 5% minimum sequence threshold. The bias vector is the same as in Figure 2 of the main text. In these simulations the most common taxa (T1) was forced to have the greatest positive bias or the greatest negative bias (low bias scenario = Low T1+ or Low T1-; high bias scenario = High T1+ and High T1-). Plots show the bias vectors and the corresponding population diet summaries are illustrated as bar plots. Numbers on top of bars are Bray-Curtis dissimilarity compared to true diet. Again, the average number of taxa per sample was 3 or 20. See Box 2 text and below for details.

## Simulation methods

-- The R-code used in simulations is available in the Dryad entry (<https://www.datadryad.org/>) associated with the paper doi:10.5061/dryad.jt07145. --

We carried out simulations assuming a population with a total of 40 food taxa in its diet, occurring in exponentially declining abundance in the environment; namely, the proportion of taxa *i* in the environment was calculated as

$$\pi_{i}=\frac{i^{-1.2}}{\sum_{i=1}^{40} i^{-1.2}}$$

We assumed a sample size of 100 scats, where the number of food taxa in each scat was generated using a binomial distribution with a mean of either 3 or 20. For a scat containing *x* food taxa, which *x* of the 40 possible was sampled in proportion to abundance in the environment.


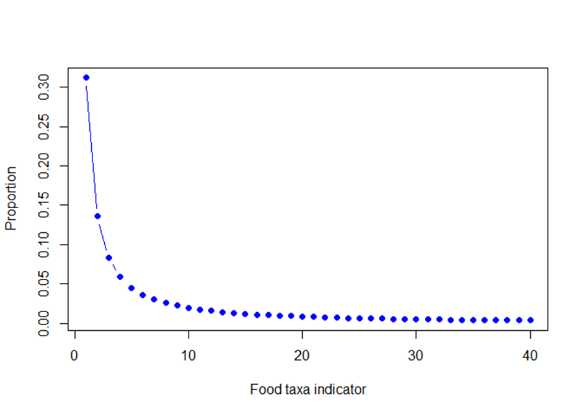


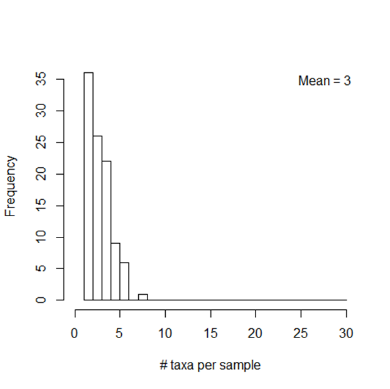

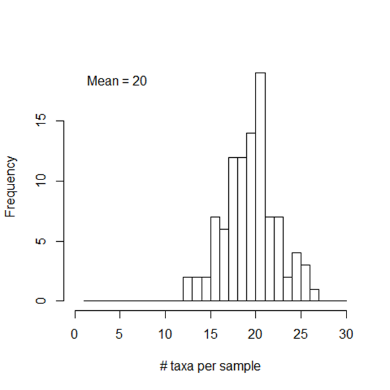


1000 sequence reads were simulated for each scat. To do so, we needed to make an assumption about the sequence recovery biases for each taxa. We define $\beta_{i}$ to be the bias factor for taxa $i$, meaning that sequences from taxa $i$ are $\beta_{i}$ times as likely to be recovered than their true proportion in the scat, relative to a standard (i.e.,$\beta$ is the relative correction factor (RCF) described in Thomas et al. 2016). Sequence counts were generated using a multinomial distribution with size 1000 and probability vector *p* (of length *x*), where *p* contains $\beta\pi$ values for the *x* taxa in the scat, rescaled to sum to 1; e.g. if a scat contains taxa 1, taxa 4 and taxa 15, then $p=\left\{ \frac{{\beta_{1}\pi}_{1}}{\theta},\frac{{\beta_{4}\pi}_{4}}{\theta},\frac{{\beta_{15}\pi}_{15}}{\theta} \right\}$ where $\theta={\beta_{1}\pi}_{1}+{\beta_{4}\pi}_{4}+{\beta_{15}\pi}_{15}$.

We considered the following bias scenarios:

- *No bias*: sequences were recovered in proportion to their abundance in the scat
- *Low bias*: sequences were recovered assuming a random bias factor for each taxa generated using a lognormal distribution with mean and standard deviation on the log scale of 0 and 0.46.
- *High bias*: sequences were recovered assuming a random bias factor for each taxa generated using a lognormal distribution with mean and standard deviation on the log scale of 0 and 1.0.

For the “No bias” scenario, $\beta_{i}=1$ for all taxa. For the “Low bias” and “High bias” scenarios, an example of the of bias factors generated for one simulation are shown in the main text (Box 2, Figure 2).

Once sequence counts were generated for the 100 samples, we estimated the proportion of each taxa in the diet using (i) RRA and (ii) POO with a minimum sequence threshold of 1% (POO 1%) (see Box 1 of main text for definitions of diet summary methods). We also calculated POO summaries using thresholds of 0.1% and 5% for comparison (Supplementary Figure S1). To evaluate how well the methods performed, we calculated the Bray-Curtis dissimilarity between the estimated and true proportions over all 40 taxa. A small complexity is that the true proportions are not equal to the proportions in the environment (i.e., the $\pi$ values), but depend on the average number of taxa in each scat. Thus, to calculate true proportions, we set the sample size to 10,000, assumed no sequence recovery biases, and calculated the diet proportions using RRA. For each average taxa per sample level (3 or 20) and bias scenario, we conducted 1000 simulations and obtained 1000 Bray-Curtis dissimilarity measures for each of the diet summary methods considered (results for RRA and POO 1% are summarized in Box 2, Figure 2 in main text).

If the sequence recovery bias is highest on the most common taxa (taxa 1), this will have the greatest effect on the results. Thus, to investigate what happens when the sequence counts for taxa 1 are highly positively biased or highly negatively biased, we conducted additional simulations in which we generated random bias vectors according to the low and high bias scenarios, then fixed the bias factor for taxa 1 at 3 SDs above and below the mean, respectively; this equates to bias factors of $exp\left( \pm3*0.46 \right)=$4 and 1/4 for the low bias scenario, and to bias factors of $exp\left( \pm3*1.0 \right)$ = 20 and 1/20 for the high bias scenario (see Box 2, Figure 2 in main text).

**Table S1.** Use of relative read abundance (RRA) and frequency of occurrence (FOO) in studies of bacterial/archaeal communities based on 16S ribosomal DNA published in Molecular Ecology since 2017. All used RRA, 11/21 used FOO.

| **Focal taxa** | **Reference** | **FOO** | **RRA** | **Marker** | **Notes** | **Article** | **Journal** | **Volume, issue, pages** |
| --- | --- | --- | --- | --- | --- | --- | --- | --- |
| Bacteria & archaea | Mestre *et al*. (2017) | N | Y | 16S |  | [Spatial variability of marine bacterial and archaeal communities along the particulate matter continuum](http://onlinelibrary.wiley.com/doi/10.1111/mec.14421/full) | MOLECULAR ECOLOGY | Volume 26, Issue 24, December 2017, Pages: 6827–6840. |
| Bacteria | Fu *et al*. (2017) | N | Y | 16S |  | [Aquatic animals promote antibiotic resistance gene dissemination in water via conjugation: Role of different regions within the zebra fish intestinal tract, and impact on fish intestinal microbiota](http://onlinelibrary.wiley.com/doi/10.1111/mec.14255/full) | MOLECULAR ECOLOGY | Volume 26, Issue 19, October 2017, Pages: 5318–5333. |
| Bacteria | Audsley *et al*. (2017) | N | Y | 16S |  | [Wolbachia infection alters the relative abundance of resident bacteria in adult Aedes aegypti mosquitoes, but not larvae](http://onlinelibrary.wiley.com/doi/10.1111/mec.14436/full) | MOLECULAR ECOLOGY | Volume 27, Issue 1, January 2018, Pages 297–309 |
| Bacteria, archaea, fungi, protists | Dassen *et al*. (2017) | N | Y | 16S & 18S | Bray-Curtis | [Differential responses of soil bacteria, fungi, archaea and protists to plant species richness and plant functional group identity](http://onlinelibrary.wiley.com/doi/10.1111/mec.14175/full) | MOLECULAR ECOLOGY | Volume 26, Issue 15, August 2017, Pages: 4085–4098. |
| Bacteria | Subirats *et al*. (2017) | N | Y | 16S | Bray-Curtis | [Wastewater pollution differently affects the antibiotic resistance gene pool and biofilm bacterial communities across streambed compartments](http://onlinelibrary.wiley.com/doi/10.1111/mec.14288/full) | MOLECULAR ECOLOGY | Volume 26, Issue 20, October 2017, Pages: 5567–5581. |
| Bacteria | Jiao *et al*. (2017) | N | Y | 16S | Bray-Curtis | [Biogeography and ecological diversity patterns of rare and abundant bacteria in oil-contaminated soils](http://onlinelibrary.wiley.com/doi/10.1111/mec.14218/full) | MOLECULAR ECOLOGY | Volume 26, Issue 19, October 2017, Pages: 5305–5317. |
| Bacteria | Quero *et al*. (2017) | N | Y | 16S | Bray-Curtis | [Seasonal rather than spatial variability drives planktonic and benthic bacterial diversity in a microtidal lagoon and the adjacent open sea](http://onlinelibrary.wiley.com/doi/10.1111/mec.14363/full) | MOLECULAR ECOLOGY | Volume 26, Issue 21, November 2017, Pages: 5961–5973. |
| Bacteria, archaea, fungi | Chen *et al*. (2017) | N | Y | 16S & ITS1 | Bray-Curtis | [Distinct microbial communities in the active and permafrost layers on the Tibetan Plateau](http://onlinelibrary.wiley.com/doi/10.1111/mec.14396/full) | MOLECULAR ECOLOGY | Volume 26, Issue 23, December 2017, Pages: 6608–6620. |
| Bacteria | Arnaud-Haond *et al*. (2017) | N | Y | 16S | Bacterial, Bray-Curtis | [Entangled fates of holobiont genomes during invasion: nested bacterial and host diversities in Caulerpa taxifolia](http://onlinelibrary.wiley.com/doi/10.1111/mec.14030/full) | MOLECULAR ECOLOGY | Volume 26, Issue 8, April 2017, Pages: 2379–2391. |
| Bacteria | Samad *et al*. (2017) | N | Y | 16S | Bray-Curtis | [Response to nitrogen addition reveals metabolic and ecological strategies of soil bacteria](http://onlinelibrary.wiley.com/doi/10.1111/mec.14275/full) | MOLECULAR ECOLOGY | Volume 26, Issue 20, October 2017, Pages: 5500–5514. |
| Bacteria | Wasimudden *et al*. (2017) | Y | Y | 16S | Weighted & unweighted UniFrac | [Gut microbiomes of free-ranging and captive Namibian cheetahs: Diversity, putative functions and occurrence of potential pathogens](http://onlinelibrary.wiley.com/doi/10.1111/mec.14278/full) | MOLECULAR ECOLOGY | Volume 26, Issue 20, October 2017, Pages: 5515–5527. |
| Bacteria | Bost *et al*. (2017) | Y | Y | 16S | Jaccard & Bray-Curtis | [How gut transcriptional function of Drosophila melanogaster varies with the presence and composition of the gut microbiota](http://onlinelibrary.wiley.com/doi/10.1111/mec.14413/full) | MOLECULAR ECOLOGY | Volume 27, Issue 8, April 2018, Pages: 1848-1859 |
| Bacteria | Feng *et al*. (2017) | Y | Y | 16S | Jaccard & Bray-Curtis | [Biodiversity and species competition regulate the resilience of microbial biofilm community](http://onlinelibrary.wiley.com/doi/10.1111/mec.14356/full) | MOLECULAR ECOLOGY | Volume 26, Issue 21, November 2017, Pages: 6170–6182. |
| Bacteria | Kropáčková *et al*. (2017) | Y | Y | 16S | Jaccard, unweighted UniFrac, Bray-Curtis | [Codiversification of gastrointestinal microbiota and phylogeny in passerines is not explained by ecological divergence](http://onlinelibrary.wiley.com/doi/10.1111/mec.14144/full) | MOLECULAR ECOLOGY | Volume 26, Issue 19, October 2017, Pages: 5292–5304. |
| Bacteria, fungi | Shukla *et al*. (2017) | Y | Y | 16S, ITS1 & 2 | Unweighted UniFrac | [Burying beetles regulate the microbiome of carcasses and use it to transmit a core microbiota to their offspring](http://onlinelibrary.wiley.com/doi/10.1111/mec.14269/full) | MOLECULAR ECOLOGY | Volume 27, Issue 8, April 2018, Pages: 1980-1991 |
| Bacteria | Nishida & Ochman (2017) | Y | Y | 16S | Unweighted UniFrac | [Rates of Gut Microbiome Divergence in Mammals](http://onlinelibrary.wiley.com/doi/10.1111/mec.14473/full) | MOLECULAR ECOLOGY | Volume 27, Issue 8, April 2018, Pages: 1884-1897 |
| Bacteria | Risely *et al*. (2017) | Y | Y | 16S | Unweighted UniFrac, Bray-Curtis, LEfSe | [Gut microbiota of a long-distance migrant demonstrates resistance against environmental microbe incursions](http://onlinelibrary.wiley.com/doi/10.1111/mec.14326/full) | MOLECULAR ECOLOGY | Volume 26, Issue 20, October 2017, Pages: 5842–5854. |
| Bacteria | Longo & Zamudio (2017) | Y | Y | 16S | Unweighted UniFrac & heatmaps | [Temperature variation, bacterial diversity and fungal infection dynamics in the amphibian skin](http://onlinelibrary.wiley.com/doi/10.1111/mec.14220/full) | MOLECULAR ECOLOGY | Volume 26, Issue 18, September 2017, Pages: 4787–4797. |
| Bacteria | Kwan *et al*. (2017) | Y | Y | 16S | Weighted & unweighted UniFrac | [Vertical vs. horizontal transmission of the microbiome in a key disease vector, Ixodes pacificus](http://onlinelibrary.wiley.com/doi/10.1111/mec.14391/full) | MOLECULAR ECOLOGY | Volume 26, Issue 23, December 2017, Pages: 6578–6589. |
| Bacteria | Ben-Yosef *et al*. (2017) | Y | Y | 16S | Sorensen index, Bray-Curtis | [Host-specific associations affect the microbiome of Philornis downsi, an introduced parasite to the Galápagos Islands](http://onlinelibrary.wiley.com/doi/10.1111/mec.14219/full) | MOLECULAR ECOLOGY | Volume 26, Issue 18, September 2017, Pages: 4644–4656. |
| Bacteria & algae | Marcelino *et al*. (2017) | Y | Y | 16S, UPA, tufA | Sorensen | [Diversity and stability of coral endolithic microbial communities at a naturally high pCO2 reef](http://onlinelibrary.wiley.com/doi/10.1111/mec.14268/full) | MOLECULAR ECOLOGY | Volume 26, Issue 19, October 2017, Pages: 5344–5357. |

**Table S2.** Use of relative read abundance (RRA) and frequency of occurrence (FOO) in metabarcoding studies of eukaryote communities published in Molecular Ecology, Molecular Ecology Resources (MER) or Methods in Ecology and Evolution (MEE) since 2016. 14/22 studies used FOO, RRA was used exclusively for eight studies (typically on fungi), most studies that didn’t use RRA (3/4) were on metazoans.

| **Focal Taxa** | **Reference** | **FOO** | **RRA** | **Marker** | **Notes** | **Article** | **Journal** | **Volume, issue, pages** |
| --- | --- | --- | --- | --- | --- | --- | --- | --- |
| Eukaryotes (inc. metazoans) | Lanzén et al. (2016) | Y | Y | 18S | Only used presence-absence to demonstrate the improvement with rel. abundance | [High-throughput metabarcoding of eukaryotic diversity for environmental monitoring of offshore oil-drilling activities](http://onlinelibrary.wiley.com/doi/10.1111/mec.13761/full) | MOLECULAR ECOLOGY | Volume 25, Issue 17, September 2016, Pages: 4392–4406. |
| Eukaryotes (inc. metazoans) | Brannock et al. (2016) | Y | Y | 18S | Jaccard & weighted UniFrac, Bray-Curtis | [Metabarcoding reveals environmental factors influencing spatio-temporal variation in pelagic micro-eukaryotes](http://onlinelibrary.wiley.com/doi/10.1111/mec.13709/full) | MOLECULAR ECOLOGY | Volume 25, Issue 15, August 2016, Pages: 3593–3604. |
| Eukaryotes (non-metazoan) | Capo et al. (2016) | Y | Y | 18S | Jaccard, Bray-Curtis | [Long-term dynamics in microbial eukaryotes communities: a palaeolimnological view based on sedimentary DNA](http://onlinelibrary.wiley.com/doi/10.1111/mec.13893/full) | MOLECULAR ECOLOGY | Volume 25, Issue 23, December 2016, Pages: 5925–5943. |
| Cercozoa | Fiore-Donno et al. (2017) | Y | Y | 18S |  | [New barcoded primers for efficient retrieval of cercozoan sequences in high-throughput environmental diversity surveys, with emphasis on worldwide biological soil crusts](http://onlinelibrary.wiley.com/doi/10.1111/1755-0998.12729/full) | MER | Volume 18, Issue 2, March 2018, Pages: 229-239. |
| Diatoms | Apothéloz-Perret-Gentil et al. (2017) | N | Y | 18S |  | [Taxonomy-free molecular diatom index for high-throughput eDNA biomonitoring](http://onlinelibrary.wiley.com/doi/10.1111/1755-0998.12668/full) | MER | Volume 17, Issue 6, November 2017, Pages: 1231–1242. |
| Fungi | Chen *et al*. (2017) | Y | N | 18S | Network analysis | [Phylogenetic relatedness explains highly interconnected and nested symbiotic networks of woody plants and arbuscular mycorrhizal fungi in a Chinese subtropical forest](http://onlinelibrary.wiley.com/doi/10.1111/mec.14061/full) | MOLECULAR ECOLOGY | Volume 26, Issue 9, May 2017, Pages: 2563–2575. |
| Fungi | Glassman et al. (2017) | Y | Y | ITS1 | Jaccard, Bray-Curtis | [Environmental filtering by pH and soil nutrients drives community assembly in fungi at fine spatial scales](http://onlinelibrary.wiley.com/doi/10.1111/mec.14414/full) | MOLECULAR ECOLOGY | Volume 26, Issue 24, December 2017, Pages: 6960–6973. |
| Fungi | Lee et al. (2017) | Y | Y | ITS1 & 2 | Presence plot | [Diversity and abundance of human-pathogenic fungi associated with pigeon faeces in urban environments](http://onlinelibrary.wiley.com/doi/10.1111/mec.14216/full) | MOLECULAR ECOLOGY | Volume 26, Issue 17, September 2017, Pages: 4574–4585. |
| Fungi | Grau et al. (2017) | Y | Y | ITS2 | Compared presence-absence and read abundance, focussed on PA due to nMDS stress, Jaccard, tested Bray-Curtis | [Abrupt changes in the composition and function of fungal communities along an environmental gradient in the high Arctic](http://onlinelibrary.wiley.com/doi/10.1111/mec.14227/full) | MOLECULAR ECOLOGY | Volume 26, Issue 18, September 2017, Pages: 4798–4810. |
| Fungi | López-García *et al*. (2017) | N | Y | 18S | Abundance-weighted data | [Plant traits determine the phylogenetic structure of arbuscular mycorrhizal fungal communities](http://onlinelibrary.wiley.com/doi/10.1111/mec.14403/full) | MOLECULAR ECOLOGY | Volume 26, Issue 24, December 2017, Pages: 6948–6959. |
| Fungi | Abrego et al. (2018) | N | Y | ITS2 |  | [Give me a sample of air and I will tell which species are found from your region – molecular identification of fungi from airborne spore samples](http://onlinelibrary.wiley.com/doi/10.1111/1755-0998.12755/full) | MER | Volume 18, Issue 3, May 2018, Pages: 511-524 |
| Fungi | Li et al. (2017) | N | Y | ITS2 | Bray-Curtis | [Highlighting patterns of fungal diversity and composition shaped by ocean currents using the East China Sea as a model](http://onlinelibrary.wiley.com/doi/10.1111/mec.14440/full) | MOLECULAR ECOLOGY | Volume 27, Issue 2, January 2018, Pages: 564-576 |
| Fungi | Vanhove et al. (2017) | N | Y | ITS2 | Bray-Curtis, LEfSe | [Genomic epidemiology of Cryptococcus yeasts identifies adaptation to environmental niches underpinning infection across an African HIV/AIDS cohort](http://onlinelibrary.wiley.com/doi/10.1111/mec.13891/full) | MOLECULAR ECOLOGY | Volume 26, Issue 7, April 2017, Pages: 1991–2005. |
| Fungi & bacteria | Gomez-Polo et al. (2017) | N | Y | 18S & 16S | Bray-Curtis | [An exceptional family: Ophiocordyceps-allied fungus dominates the microbiome of soft scale insects (Hemiptera: Sternorrhyncha: Coccidae)](http://onlinelibrary.wiley.com/doi/10.1111/mec.14332/full) | MOLECULAR ECOLOGY | Volume 26, Issue 20, October 2017, Pages: 5855–5868. |
| Fungi & bacteria | Xiao et al. (2017) | N | Y | ITS1 & 16S | Bray-Curtis | [Fungal community reveals less dispersal limitation and potentially more connected network than that of bacteria in bamboo forest soils](http://onlinelibrary.wiley.com/doi/10.1111/mec.14428/full) | MOLECULAR ECOLOGY | Volume 27, Issue 2, January 2018, Pages: 550-563 |
| Fungi & bacteria | Bordez et al. (2016) | N | Y | ITS2 & 16S | Bray-Curtis | [Distribution patterns of microbial communities in ultramafic landscape: a metagenetic approach highlights the strong relationships between diversity and environmental traits](http://onlinelibrary.wiley.com/doi/10.1111/mec.13621/full) | MOLECULAR ECOLOGY | Volume 25, Issue 10, May 2016, Pages: 2258–2272. |
| Invertebrates | Carew *et al*. (2017) | Y | N | COI | Degraded DNA | [Detecting invertebrate species in archived collections using next-generation sequencing](http://onlinelibrary.wiley.com/doi/10.1111/1755-0998.12644/full) | MER | Volume 17, Issue 5, September 2017, Pages: 915–930. |
| Invertebrates | Andújar *et al*. (2018) | Y | N | 18S & COI | Jaccard/Sorensen | [Metabarcoding of freshwater invertebrates to detect the effects of a pesticide spill](http://onlinelibrary.wiley.com/doi/10.1111/mec.14410/full) | MOLECULAR ECOLOGY | Volume 27, Issue 1, January 2018, Pages 146–166. |
| Invertebrates | Elbrecht et al. (2017) | Y | Y | COI |  | [Assessing strengths and weaknesses of DNA metabarcoding‐based macroinvertebrate identification for routine stream monitoring](http://onlinelibrary.wiley.com/doi/10.1111/2041-210X.12789/full) | MEE | Volume 8, Issue 10, October 2017, Pages 1265–1275 |
| Mosquitoes & Ross River virus | Batovska et al. (2018) | Y | Y | COI & RRV E2 | Species survey | [Effective mosquito and arbovirus surveillance using metabarcoding](http://onlinelibrary.wiley.com/doi/10.1111/1755-0998.12682/full) | MER | Volume 18, Issue 1, January 2018, Pages 32–40 |
| Mammals | Rodgers *et al*. (2017) | Y | N | 16S & 12S | Species survey | [Carrion fly-derived DNA metabarcoding is an effective tool for mammal surveys: Evidence from a known tropical mammal community](http://onlinelibrary.wiley.com/doi/10.1111/1755-0998.12701/full) | MER | Volume 17, Issue 6, November 2017, Pages: e133–e145. |
| Frogs | Lopes et al. (2017) | Y | Y | 12S | Species survey, correlation w' no. reads | [eDNA metabarcoding: a promising method for anuran surveys in highly diverse tropical forests](http://onlinelibrary.wiley.com/doi/10.1111/1755-0998.12643/full) | MER | Volume 17, Issue 5, September 2017, Pages: 904–914. |
